# *In-vitro* assessment of cutaneous immune responses to *aedes* mosquito salivary gland extract and dengue virus in Cambodian individuals

**DOI:** 10.1093/oxfimm/iqae003

**Published:** 2024-04-01

**Authors:** David Guerrero, Sokchea Lay, Eakpor Piv, Chansophea Chhin, Sokkeang Leng, Ratana Meng, Kim Eng Mam, Polidy Pean, Amelie Vantaux, Sebastien Boyer, Dorothée Missé, Tineke Cantaert

**Affiliations:** Institut Pasteur du Cambodge, Immunology Unit, Pasteur Network, Phnom Penh 12201, Cambodia; Institut Pasteur du Cambodge, Immunology Unit, Pasteur Network, Phnom Penh 12201, Cambodia; Institut Pasteur du Cambodge, Malaria Unit, Pasteur Network, Phnom Penh 12201, Cambodia; Institut Pasteur du Cambodge, Malaria Unit, Pasteur Network, Phnom Penh 12201, Cambodia; Institut Pasteur du Cambodge, Medical and Veterinary Entomology Unit, Phnom Penh 12201, Cambodia; Institut Pasteur du Cambodge, Immunology Unit, Pasteur Network, Phnom Penh 12201, Cambodia; Crystal Esthetic Center, Phnom Penh 12201, Cambodia; Institut Pasteur du Cambodge, Immunology Unit, Pasteur Network, Phnom Penh 12201, Cambodia; Institut Pasteur du Cambodge, Malaria Unit, Pasteur Network, Phnom Penh 12201, Cambodia; Institut Pasteur du Cambodge, Medical and Veterinary Entomology Unit, Phnom Penh 12201, Cambodia; Unité Ecologie et Emergence des Pathogènes Transmis par les Arthropodes, Institut Pasteur, Paris, France; MIVEGEC, Univ. Montpellier, IRD, CNRS, 34000, Montpellier, France; Institut Pasteur du Cambodge, Immunology Unit, Pasteur Network, Phnom Penh 12201, Cambodia

**Keywords:** skin immunity, dengue, *Aedes* mosquito, arboviruses, salivary gland extract

## Abstract

Dengue virus (DENV) poses a global health threat, affecting millions individuals annually with no specific therapy and limited vaccines. Mosquitoes, mainly *Aedes aegypti* and *Aedes albopictus* worldwide, transmit DENV through their saliva during blood meals. In this study, we aimed to understand how *Aedes* mosquito saliva modulate skin immune responses during DENV infection in individuals living in mosquito-endemic regions. To accomplish this, we dissociated skin cells from Cambodian volunteers and incubated them with salivary gland extract (SGE) from three different mosquito strains: *Ae. aegypti* USDA strain, *Ae. aegypti* and *Ae. albopictus* wild type (WT) in the presence/absence of DENV. We observed notable alterations in skin immune cell phenotypes subsequent to exposure to *Aedes* salivary gland extract (SGE). Specifically, exposure lead to an increase in the frequency of macrophages expressing chemokine receptor CCR2, and neutrophils expressing CD69. Additionally, we noted a substantial increase in the percentage of macrophages that became infected with DENV in the presence of *Aedes* SGE. Differences in cellular responses were observed when *Aedes* SGE of three distinct mosquito strains were compared. Our findings deepen the understanding of mosquito saliva's role in DENV infection and skin immune responses in individuals regularly exposed to mosquito bites. This study provides insights into skin immune cell dynamics that could guide strategies to mitigate DENV transmission and other arbovirus diseases.

## Introduction

Dengue virus (DENV) is a globally widespread flavivirus that affects approximately 400 million people annually. Out of those infected, around 96 million develop symptoms, with 2 million of these symptomatic cases developing severe manifestations of the disease, and approximately 20 000 cases resulting in fatalities [1]. At present, there is no therapy available for dengue fever, and although there are two approved vaccines, one has restricted use for individuals with laboratory-confirmed previous dengue infection, while the other is approved in the European Union, Indonesia and Brazil and remains under further evaluation [2]. In recent years, there has been a notable increase in the incidence and intensity of outbreaks associated with mosquito-borne viruses. Climate change plays a pivotal role in driving these epidemiological shifts [3, 4]. Indeed, climate change facilitates the settling of insect vectors into new regions and, in doing so, creates novel ecological niches favorable to their proliferation [5, 6]. Furthermore, different aspects of both mosquitoes and viruses are influenced by climate—mosquitoes’ longevity, biting rate and fecundity as well as the virus extrinsic incubation period are influenced by temperature, and higher temperatures are associated with higher transmission [7]. Mosquitoes, which are the most notorious arbovirus vectors, take up arboviruses by inserting their proboscis into the dermis of an infected host during a blood meal. The virus then replicates in midgut cells before disseminating and reaching the salivary glands. During a subsequent blood meal, the infectious mosquito injects the virus and insect-derived molecules, including salivary components, into the epidermis and dermis of the human host [8]. Each of these skin layers harbors a diverse array of specialized cell types. These cell populations include keratinocytes, Langerhans cells (LCs), gamma-delta (γδ) T cells, resident memory T cells (TRM), fibroblasts, macrophages, dendritic cells, and mast cells. These cells collectively play pivotal roles in the immunological defense mechanisms of the human body, contributing to the maintenance of tolerance towards beneficial commensal microorganisms and to the orchestrated immune response against pathogenic challenges.

Skin-resident immune cells possess the capacity to detect pathogen-associated molecular patterns (PAMPs) through pattern recognition receptors (PRRs). Upon activation, these immune cells initiate the secretion of various cytokines and chemokines making them a first line of defense against external pathogenic insults [9].

Saliva from hematophagous mosquitoes is a cocktail of pharmacologically active molecules, some of which are anti-hemostatic, angiogenic, and immunomodulatory [10–12]. Importantly, the expression of salivary factors can differ based on the mosquito’s feeding status (blood-fed or unfed) as well as its infection status [13, 14]. Over the past few decades, several animal and *in vitro* studies have shown that inoculation of vector saliva, and/or concomitant blood-feeding by an arthropod, can modulate the host response to pathogens in the skin and periphery [12]. These modulations result in altered cytokine production profiles [15, 16], promotion of recruitment of infection-susceptible cells to the bite site [17], activation of autophagy [18], and an induction of neutrophil infiltration to the local inflammation site [19], among other consequences. Overall, it is well accepted that arthropod-mediated viral infection leads to increased disease progression, viremia, and mortality [12, 20].

While *Ae. aegypti* and *Ae. albopictus* are both capable of transmitting DENV, *Ae. aegypti* is considered the primary and more efficient vector of the two [21, 22]. This has resulted in less attention being given to the study of secondary vectors, such as *Ae. albopictus*, and their potential role in skin immunity and arbovirus disease pathogenesis. Moreover, studies comparing the effect of both mosquito strains are rare. Similarly, there has been a bias towards using laboratory-reared *Aedes* mosquito strains in experimental settings, leading to a scarcity of investigations on the effectof WT *Aedes* strains saliva on the modulation of the host response to arboviruses. Moreover, in spite of the compelling data derived from animal and *in vitro* research, there are little translational research studies concerning the immune response of human skin cells against viral infection in the presence of mosquito saliva. This gap in knowledge is particularly pronounced with regards to human populations in tropical regions, where individuals sustain persistent exposure to mosquito bites. This chronic exposure potentially causes distinct immune reactions, diverging from those observed in individuals subject to sporadic or infrequent bites [23–25]. Indeed, in a study conducted by Pingen and collaborators, it was observed that pre-exposure of mice to mosquito bites primed them to rapidly express cutaneous IFN-γ and IL-10 upon further mosquito biting [19].

Here, we attempted to gain a more profound understanding of the effect generated by DENV and saliva of different *Aedes* mosquitoes (*Ae. aegypti* USDA strain, *Ae. aegypti* and *Ae. albopictus* WT—F1–3) on the local cutaneous immune response. Our interest focused particularly on individuals living in *Aedes* and DENV endemic regions. We evaluated alterations in the profiles of activation and differentiation markers from skin immune cells exposed to SGE in the presence or absence of DENV. Moreover, we aimed to assess differences in DENV infection of innate skin immune cells when concomitantly exposed to SGE. We found that exposure to *Aedes* mosquito saliva caused notable alterations in the phenotypes of immune skin cells. This included a moderate increase in macrophages expressing the CCR2 marker, higher proportions of neutrophils expressing CD69, and a substantial rise in macrophages infection rates. Additionally, we observed differences in the immunomodulatory capacities of the SGE from the different *Aedes* strains.

## Results

### Salivary gland extract from *Ae. aegypti*, but not *Ae. albopictus* has an effect on migration and activation markers in skin immune cells

The study group consisted of 32 individuals, all of whom were female of Khmer origin, with a median age of 36 years [29.75–41.00].

**Figure 1.**
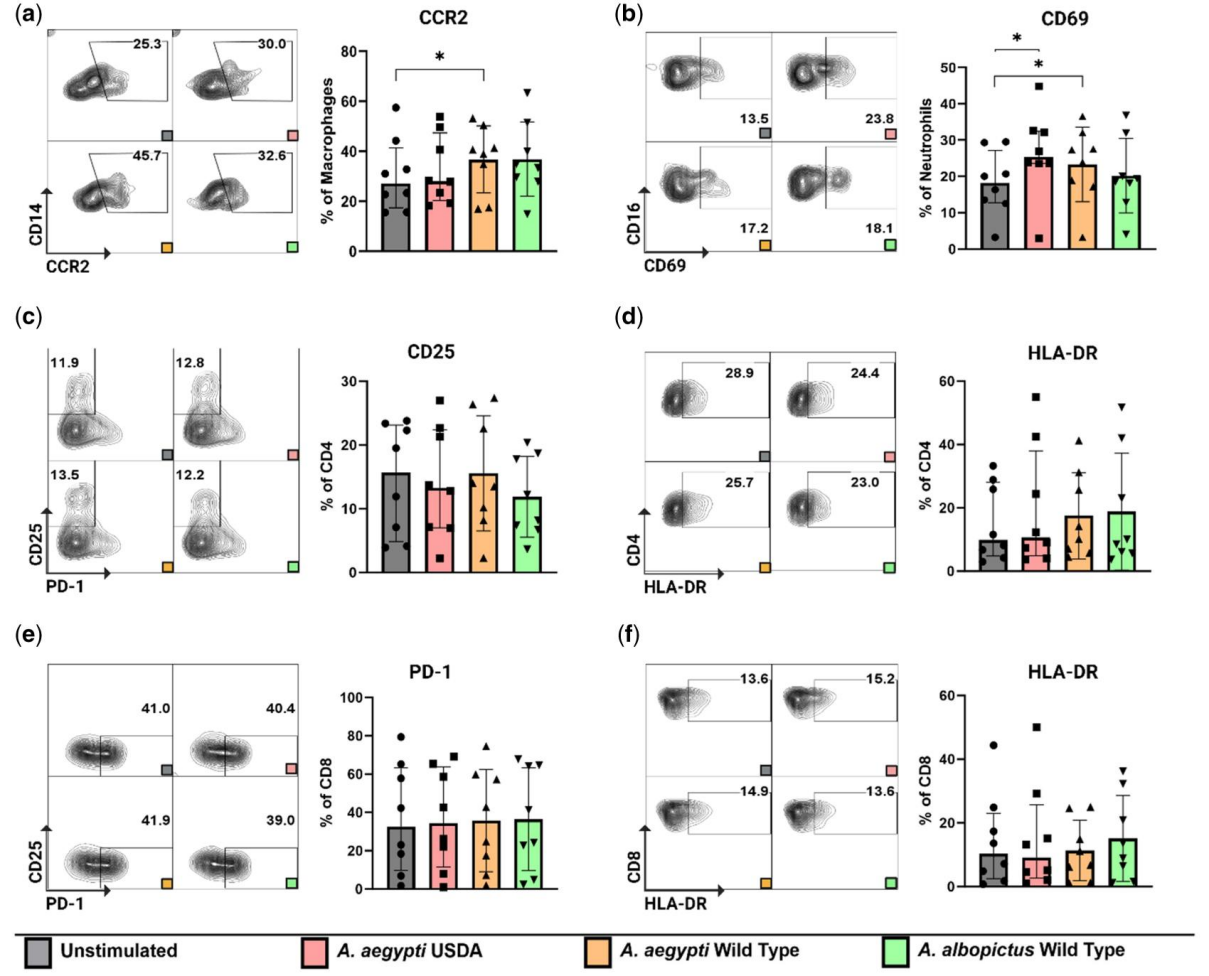
Activation marker expression on the innate immune cells and the T cell compartment after SGE treatment. Cells were exposed to SGE for 48 h. (**a**) Increased proportion of macrophages (CD45^+^ CD11b^+^CD14^+^) expressing CCR2 after being treated with SGE from *Ae. aegypti* WT. (**b**) Increased proportions of neutrophils (CD45^+^ CD15^+^CD16^+^) expressing CD69 after being treated with SGE from *Ae. aegypti* USDA and WT strains. (**c and d**) No significant change in the expression of activation markers CD25 and HLA-DR on CD4^+^ T cells. (**e and f**) No significant difference in the expression of activation markers PD-1 and HLA-DR on CD8^+^ T cells. Statistical analysis were performed with Wilcoxon signed-rank test two tailed comparing the unstimulated condition to each of the different stimulated conditions. Bars indicate median and interquartile range. Gray: unstimulated condition, Red: SGE from *Ae. aegypti* USDA strain, Yellow: SGE from *Ae. aegypti* WT strain, Green: SGE from *Ae. albopictus* WT strain. *N* = 8 individuals. *P < 0.05.

Our initial interest was directed towards assessing the impact of SGE on dissociated cells derived from resting skin. To accomplish this, we exposed isolated cells to SGE at a concentration of 10 µg/ml for 48 h. After this, we stained the cells and assessed activation and migratory markers. The marker selection was based on previous experiments involving *Aedes* mosquito-bitten skin [15]. We observed a significant increase in the percentage of macrophages (CD45^+^CD11b^+^CD14^+^) expressing surface marker CCR2 when exposed to *Ae. aegypti* WT SGE. Interestingly, a trend was observed for CCR2 expression in macrophages treated with SGE prepared from *Ae. albopictus*, but significance was not achieved (*P* = 0.19). Additionally, we saw an increased proportion of neutrophils (CD45^+^CD15^+^CD16^+^) manifesting the early activation marker CD69, when treated with *Ae. aegypti* USDA and *Ae. aegypti* WT strains SGE (Fig. 1a and b). It is noteworthy that other cell types, specifically dendritic cells (characterized as CD45^+^ CD1c^+^) and CD16^+^ macrophages (CD45^+^ CD14^+^ CD16^+^) did not exhibit any appreciable alterations in the expression levels of these aforementioned markers (Supplementary Fig. 1). Turning our attention to the T cell subset, our analysis revealed no discernible alterations in the expression of activation markers such as CD25 or HLA-DR and the activation/exhaustion marker PD-1, neither in CD4^+^ nor CD8^+^ T cells after SGE incubation (Fig. 1c–f). Taken together, incubation of dissociated skin cells with SGE leads to changes in the expression of activation and migratory markers mainly in innate immune cell populations in the skin.

### *Ae. aegypti* SGE treatment leads to increased proportion of infected macrophages

**Figure 2.**
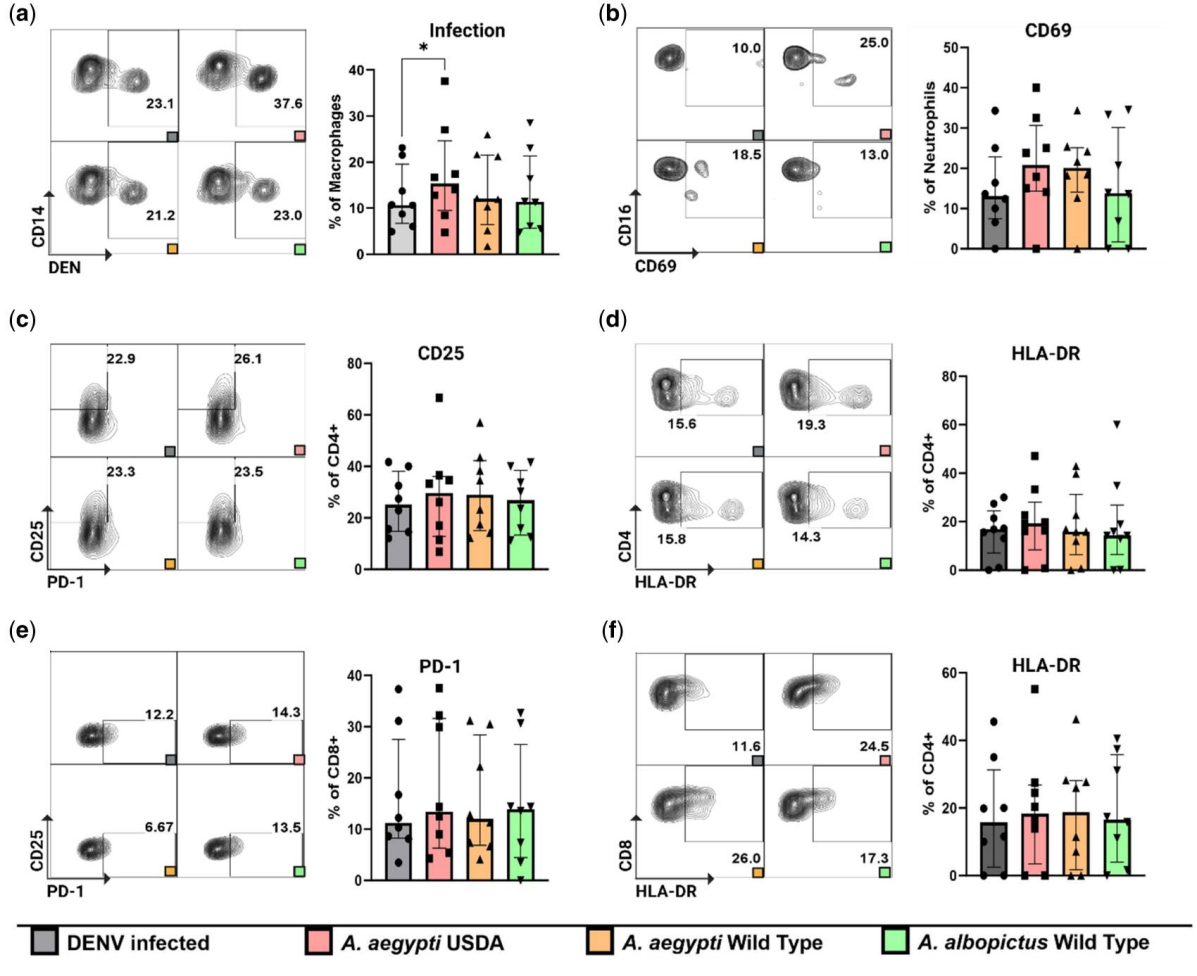
Activation marker expression on the innate immune cells and the T cell compartment after SGE treatment and DENV infection. Cells were exposed to SGE + DENV for 48 h. (**a**) Increased proportion of infected macrophages (CD45^+^ CD11b^+^CD14^+^) after being treated with SGE from *Ae. aegypti* USDA strain. No significant changes in (**b**) neutrophils (CD45^+^ CD15^+^CD16^+^) expressing CD69, (**c and d**) CD25 and HLA-DR expression on CD4^+^ T cells, and (**e and f**) PD-1 and HLA-DR expression on CD8^+^ T cells. Statistical analysis were performed with Wilcoxon signed-rank test two tailed comparing the unstimulated condition to each of the different stimulated conditions. Gray: unstimulated condition, Red: SGE from *Ae. aegypti* USDA strain, Yellow: SGE from *Ae. aegypti* WT strain, Green: SGE from *Ae. albopictus* WT strain. *N* = 8 individuals. *P < 0.05.

Next, we explored potential synergistic effects arising from the simultaneous exposure of cells to SGE and DENV. To investigate this, we subjected cells to a combination treatment comprising SGE at a concentration of 10 µg/ml and DENV2 at a MOI of 3, maintaining this coculture for a duration of 48 h. We observed a significant increase in the proportion of infected macrophages (CD45^+^ CD11b^+^CD14^+^) when they were subjected to treatment with SGE derived from the *Ae. aegypti* USDA strain compared to the control infection condition without the addition of SGE (Fig. 2a). No statistically significant variations in the expression of activation markers within the innate cell population were detected, nor did we discern any noteworthy differences within the T cell compartment (Fig. 2b–f).

### Distinct immunomodulatory effect magnitude between *Ae. aegypti* USDA strain—*Ae*. *aegypti* WT and *Ae. albopictus* WT

**Figure 3.**
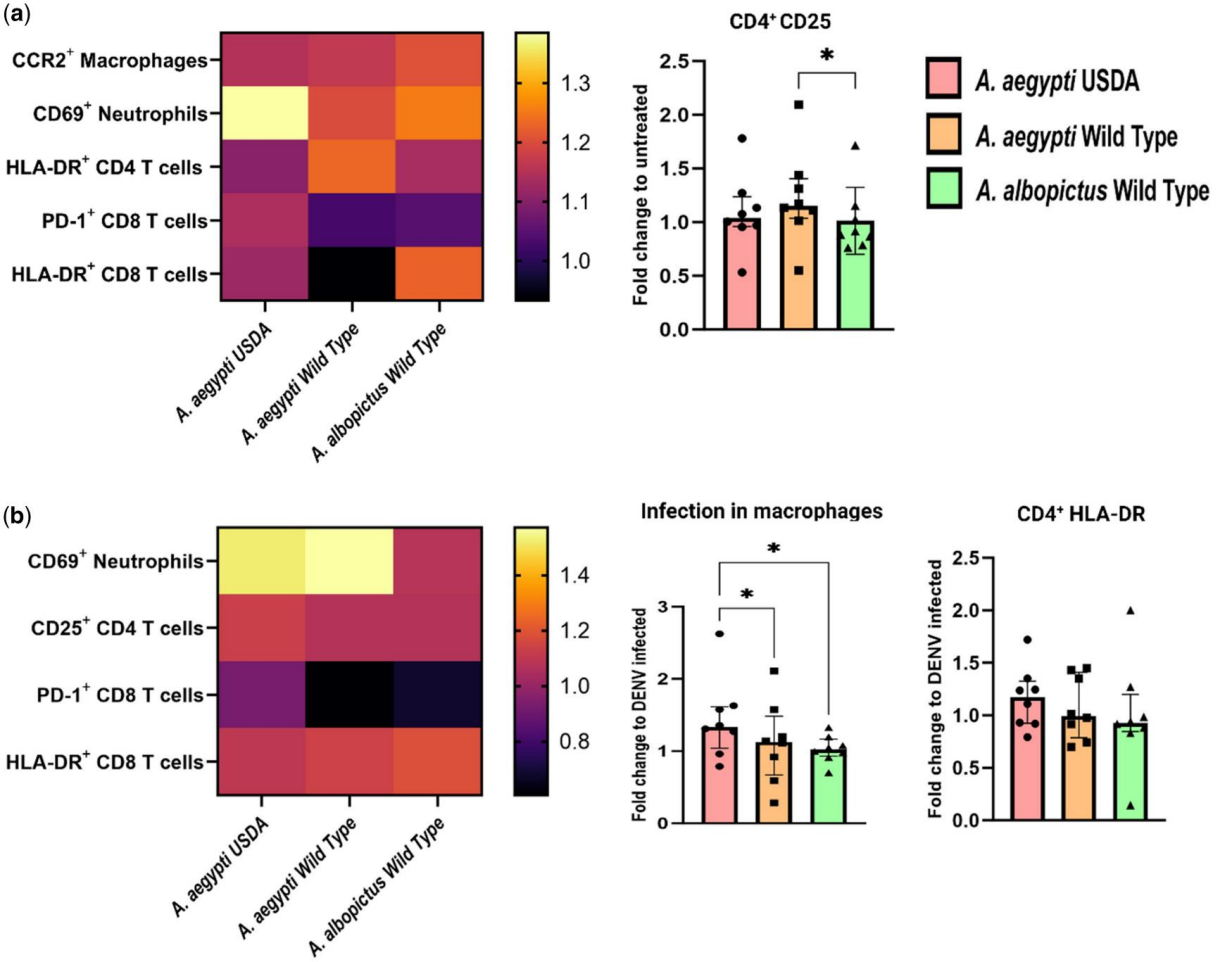
Comparative analysis of the fold change in activation markers elicited by the different SGEs. Additional analysis of the data obtained in Figs 1 and 2, showing the fold change of differences comparing the three mosquito groups between them. (**a**) Left panel: Heat map of fold change in marker expression after SGE exposure. Right panel: Higher fold change of CD4^+^ T cells expressing CD25 in conditions treated with *Ae. aegypti* WT when compared to *Ae. albopictus* WT. (**b**) Left panel: Heat map of fold change in marker expression after SGE and DENV exposure. Right panel: Increase in the fold change of infected macrophages (CD45^+^ CD11b^+^CD14^+^) when comparing *Ae. aegypti* USDA and WT SGEs. Statistical analysis were performed Friedman + Dunn’s multiple comparisons test, two tailed. Bars indicate median and interquartile range. Gray: unstimulated condition, Red: SGE from *Ae. aegypti* USDA strain, Yellow: SGE from *Ae. aegypti* WT strain, Green: SGE from *Ae. albopictus* WT strain. *N* = 8 individuals. *P < 0.05.

Following the assessment of the phenotypic alterations observed in cutaneous immune cells in the presence or absence of SGE, our focus shifted towards exploring potential subtle distinctions in the immunomodulatory capacities of SGE derived from distinct mosquito strains. Therefore, we analyzed the fold change differences in the frequency of cells expressing activation markers in the various conditions tested. Our analyses revealed that the fold change in the frequency of cells expressing activation markers within both skin innate immune cells and T lymphocytes displayed a consistent pattern across the various SGEs, with no statistically significant elevation in most instances. However, a notable exception was evident in the context of CD25 expression on CD4^+^ T cells, where CD25 is significantly upregulated in CD4^+^ T cells exposed to SGE obtained from *Ae. aegypti* WT compared to exposure to SGE from *Ae. albopictus* WT (Fig. 3a).

Using the same analytical approach, we assessed the fold changes in cells infected with DENV and in the presence of SGE obtained from the three different mosquito strains. We observed that the fold change of macrophage infection was higher in cells treated with SGE obtained from *Ae. aegypti* USDA compared to the other two strains (Fig. 3b).

Consistent with our previous analyses, we noted minimal variability in the fold change of most activation markers within both skin innate immune cells and T cell populations. It is worth noting that, although statistical significance was not achieved, there was a trend of increased HLA-DR expression on CD4^+^ T cells when subjected to treatment with *Ae. aegypti* USDA SGE (Fig. 3b).

## Discussion

The skin is the primary organ in contact with DENV after infection, and natural infection always occurs in the presence of mosquito saliva. The composition of the saliva is highly dependent on the mosquito species, mosquito feeding status and others parameters [13, 14]. Therefore, our goal was to understand how the skin immune cells from individuals living in *Aedes* and DENV endemic regions responded to saliva inoculation obtained from *Aedes* mosquitoes of different origin in the context of DENV infection. One limitation of the study is the inclusion of only individuals of the female sex, due to our skin samples originating from elective surgery. Indeed, substantial and well-documented sex-driven differences exist in the immune system [26–28]. One further aspect to consider in this study is the use of SGE. Although SGE serves as a valuable surrogate [25], its composition could differ from that of naturally secreted mosquito saliva. These distinctions underscore the need for cautious interpretation of experimental results.

We observed that exposure to SGE from WT *Ae. aegypti* mosquitoes led to an increase in the percentage of macrophages expressing the surface marker CCR2. CCR2, a chemokine receptor expressed within the monocyte and macrophage populations, plays a pivotal role in the orchestration of immune responses. The interaction with its ligands is closely associated with the recruitment of these immune cells to sites of inflammation [29, 30]. Consequently, our observation of an augmented proportion of macrophages expressing CCR2 following exposure to WT *Ae. aegypti* SGE suggests a plausible scenario wherein an elevated influx of these cells migrate to the actual bite site in a natural setting. Indeed, studies have described the capacity of salivary factors/bites from *Aedes* mosquitoes in augmenting the trafficking of monocytes to the bite site [31]. Some proposed explanations of such processes have assumed a potential regulatory role of neutrophils in orchestrating myeloid cell influx, as shown in murine models of mosquito bites [19]. Previously, we conducted an *in vivo* study in humans of the immune responses triggered by the exposure of skin to uninfected *Aedes* WT mosquito bites. Here, we observed an increase in pathways and genes related to neutrophil activation and degranulation 4 h post-bite in Cambodian volunteers [15]. In line with these findings, we observed an increase in the expression of CD69 in neutrophils in the current study, which indicates activation of these cells. Therefore, we can hypothesize that the activated neutrophils are releasing inflammatory mediators, including CCL2, to attract CCR2-expressing monocytes [32]. In natural DENV infection, an increase of these type of cells to the bite site could translate to higher viral replication as they are highly permissive to the virus [33].

Interestingly, our investigation revealed a notable increase in the proportion of DENV-infected macrophages subsequent to exposure to SGE isolated from *Ae. aegypti* USDA. This phenomenon aligns with well-documented findings in the literature, where augmented replication and susceptibility of target cells has been recurrently attributed to the influence of mosquito salivary factors. Notably, a salivary-specific protein, denominated *Ae. aegypti* venom allergen-1, induces autophagy in monocytic cells, promoting enhanced infection and transmission of both DENV and ZIKV [18]. Likewise, other authors have reported a significant increase of viral replication in keratinocytes infected with West Nile virus (WNV) in the presence of *Ae. aegypti* saliva [34]. We observed an increased proportion of infected macrophages in the presence of SGE derived from *Ae. aegypti* USDA strain but not SGE obtained from WT mosquitos. This consideration should be taken into account for future studies, where the use of SGE from WT mosquitos should be favored, if possible. Difference might be due to the fact that people are not exposed to laboratory-reared mosquitos and hence might react differently to salivary factors they have not encountered before compared to those they have been consistently exposed to (i.e. salivary proteins from WT mosquitoes). Alternatively, it is important to acknowledge that the microbiome composition within SGE may vary between WT and laboratory-reared mosquitoes. Further studies are necessary to understand these differences.

We observed an increase in the fold change of CD25 expression on CD4^+^ T cells in skin immune cells treated with SGE from WT *Ae. aegypti* in comparison to SGE from the WT *Ae. albopictus*. Moreover, we saw an increasing trend in the fold change of the expression of HLA-DR on CD4^+^ T cells when cells were treated with SGE from the *Ae. aegypti* USDA stain relative to those treated with SGE from the WT strain. Interestingly, when we performed our *Aedes* bite model study in human volunteers, HLA-DR transcripts were upregulated 48 h after the bite, together with a skewing to Th2-driven responses and CD8^+^ T cell activation [15].

Certain observed phenotypic differences were specific to particular strains (i.e. noticeable with SGE treatment obtained from one strain but not with SGE obtained from the other two strains). However, due to the limited numbers of patients available for each comparison, some phenotypic markers seem to be differentially expressed but the comparison did not reach significance. We encountered a similar constraint due to the limited availability of primary human skin cells, necessitating the selection of a single time point as read out for the stimulation assays. This limitation may have impeded our ability to observe transient changes in some markers occurring prior to our 48h time point.

Additionally, It’s important to recognize that our study was carried out in an *in vitro* environment, making it impossible to evaluate the potential impact of cell migration [15, 19]. This constrained our ability to examine the consequences arising from interactions between resident cells and circulating cells, which typically migrate to the site of inflammation during natural infection. Indeed, the introduction of the virus through either mosquito bite or simultaneous inoculation with the vector's salivary components results in an augmentation of viremia, viral replication, and dissemination in animal models [19, 35–37]. In the context of natural DENV infection, an increase in the presence of macrophages and dendritic cells at the site of the mosquito bite leads to enhanced viral replication since these cells are susceptible to DENV infection [19, 38].

In summary, this study revealed various changes in cell responses when exposed to different sources of mosquito SGE and DENV infection, providing insights into the immune reactions in the context of mosquito-borne infections.

## Materials and methods

### Patient inclusions

This study was approved by the National Ethics Committee for Health Research in Cambodia (NECHR approval no. 2020-256). The study group consisted of 32 individuals, all of whom were female of Khmer origin, with a median age of 36 years who were undergoing elective eye-lid surgery. The first set of experiments, involving SGE challenge on resting skin cells, utilized 16 individuals. An additional 16 individuals were employed for the second set of experiments, focusing on SGE treatment on DENV-infected cells.

### Skin biopsy dissociation

Leftover cutaneous tegument specimens from eye-lid aesthetical surgery were used to isolate primary human skin cells and adjacent immune cells. The specimen were transported in RPMI media (Sigma-Aldrich—Cat# R1145) on ice. Nine 4 mm biopsy punches per individual were obtained from the skin tissue specimen. A whole skin dissociation kit (Miltenyi Biotech^®^ - Cat# 130-101-540) was used to perform the skin tissue dissociation within 24 h of operation. Briefly, an enzyme mix consisting of enzyme D, enzyme A, and buffer L was prepared following the manufacturer’s specifications. Single 4 mm punch biopsies were incubated in 460 μl of dissociation mix, at 37°C and 5% CO_2_ for 6 h. Following the incubation, each sample mix was transferred to a Falcon tube with a cell-strainer cap (40–75 µm). Using a rubber pestle and the cell-strainer cap as a mortar, skin biopsies were gently macerated to increase cell recovery yield. 500 μl of cold RMPI was used to rinse caps. Dissociated cells from each skin biopsy of the same individual were then pooled and centrifuged at 428 × *g*, 4°C for 10 min. The cell pellet was resuspended in 5 ml of cold RPMI. Dissociated skin cells were manually counted using a Malassez counting chamber.

### Mosquito rearing

Female mosquitoes were reared in standard conditions of humidity 60–75% at a temperature of 27 ± 2°C. Three strains of mosquitoes were used in this study: *Ae. aegypti* USDA strain, *Ae. aegypti* WT collected in the city of Phnom Penh and *Aedes* albopictus WT also collected in Phnom Penh [39]. Collected WT mosquitoes were reared in the laboratory. Upon oviposition eggs were captured on paper strips and hatch in water trays when needed—F1, F2 and F3 WT mosquitoes were used in the experiments 15 + days post emergence. To ensure that the saliva composition closely resembled that of natural mosquito biting events, all mosquitoes were blood-fed on mice 24–48 h before collection for salivary gland dissection [13].

### *Aedes* salivary gland extract preparation

The dissection of salivary glands and the subsequent preparation of SGE were executed in accordance with the following protocol: 100 female mosquitoes were collected in paper cups and euthanized by exposure to 70% ethanol. A brief additional immersion in alcohol was performed for sterilization. Post-sterilization, the mosquitoes were subjected to a thorough rinse utilizing PBS 1× to eliminate residual alcohol traces.

The mosquitoes, subsequently positioned upon glass slides beneath a stereomicroscope, underwent a procedure involving the careful detachment of their heads from their thoracic segments. This separation exposed the salivary glands, which were then carefully harvested and pooled in low binding centrifuge tubes containing 200 μl of PBS 1× solution on ice. Later homogenization was done for 30 s at 6000 rpm on a Magnalizer bead homogenizer. The resulting solution was centrifuged at 428 × *g*, 4°C for 10 min. supernatants were collected and its SGE protein concentrations quantified using a NanoDrop spectrophotometer (*Ae. albopictus* WT 570 μg/ml, *Ae. aegypti* USDA 990 μg/ml, *Ae. aegypti* WT 1030 μg/ml), aliquoted and stored at −80°C until further use.

### *In-vitro* treatment of dissociated skin cells with SGE

A suspension of 600 000 skin dissociated cells in 200 μl of RPMI media containing 1% penicillin (Gibco), 1% glutamate (Invitrogen), and 5% fetal bovine serum (FBS, Gibco) was seeded into U-bottom 96-well plates and was incubated with SGE at a concentration of 10 μg/ml from either *Ae. aegypti* USDA strain, *Ae. aegypti* or *Ae. albopictus* WT (F1–3) for 48 h in a 37°C, 5% CO_2_ environment. Untreated cells were used as negative controls. After incubation, cells were washed with PBS 1× (428 × *g*, 4°C for 5 min) and prepared for flow cytometry staining.

### Infection with DENV2 and SGE treatment

DENV2 New Guinea C strain (GenBank: AF038403) was produced under BSL2 safety conditions as previously reported [40]. A total of 8 × 10^6^  *Aedes*  *albopictus* C6/36 cell line were placed in a 75 cm^2^ flask and allowed to grow overnight at a temperature of 28°C. Cells were then infected with the virus at a Multiplicity of Infection (MOI) of 0.1 and cultured for 5–7 days at the same temperature in Leibovitz 15 medium (Sigma-Aldrich), supplemented with 2% FBS (Gibco), 1% L-glutamine (Invitrogen), 10% tryptose-phosphate (Gibco), and 100 U/ml penicillin with 100 µg/ml streptomycin (Gibco). After this incubation period, the virus culture supernatants were collected and concentrated using a 40% polyethylene glycol (PEG) 8000 solution (Sigma-Aldrich). The concentrated virus was then suspended in FBS and stored at −80°C for future use.

For the infection, DENV2 was seeded into U-bottom 96-well plates at a MOI of 3. Simultaneously, SGE was added to the wells at a final concentration of 10 µg/ml. This co-culture was allowed to occur for a duration of 5 min at room temperature, facilitating potential interactions between the DENV particles and the constituents of the salivary extract. Subsequent to this interaction period, a suspension of 600 000 skin-dissociated cells was introduced into each well, and these cells were maintained in RPMI media containing 1% penicillin (Gibco), 1% glutamate (Invitrogen), and 5% FBS (Gibco). The co-culture of cells, DENV, and SGE was maintained at 37°C with a 5% CO_2_ environment for a duration of 48 h.

### Flow cytometry

Cells were transferred to V-bottom 96-well plates and washed with 200 μl of PBS buffer 1× (428 × *g*, 4°C for 5 min). 20 μl of Zombie Aqua fixable viability stain (Biolegend, 1 : 500) was added and incubated for 20 min in the dark at 4°C. Excess viability stain was washed away with 200 μl of PBS/BSA/EDTA buffer (428 × *g*, 4°C for 5 min) and the cell pellet resuspended and incubated for 10 min at 4°C in 4 μl of FcR blocking antibody (Biolegend) (1/10). Cells were then incubated for 30 min at 4°C with surface staining antibody master mix (Supplementary Table 1). After washing free unbound antibody with 200 μl of PBS (428 × *g*, 4°C for 5 min), cells were fixed with 100 μl fixation buffer (Biolegend) 1× for 20 min at 4°C in the dark. Following fixation, cells were permeabilized and washed with 100 μl of perm-wash solution 1× (Biolegend, 428 × *g*, 4°C for 5 min) and the cell pellet resuspended and incubated for 30 min at 4°C with anti-DENV envelop protein antibody (clone 4G2 ATCC HB-112, labeled with Alexa Fluor 488) (Molecular probes; Thermo Fisher) (antibody dilution 1/100). Stained cells were resuspended in 200 μl of PBS/BSA/EDTA 1× and visualized on a BD Biosciences FACS Aria II using FACS Diva software. Analysis was performed using BD Biosciences FlowJo x10.0.7r2 software. Gating strategy is described in Supplementary Fig. 2.

### Statistics

All data was analyzed with GraphPad’s Prism 9 software, using nonparametric-based tests: Wilcoxon test to compare baseline and SGE treatments, and Friedman + Dunn’s multiple comparisons test to compare the three different mosquito strains SGEs between each other, two tailed where applicable. Bars indicate median and interquartile range. A *P* < 0.05 was considered statistically significant.

## Supplementary Material

iqae003_Supplementary_Data

## Data Availability

All data are contained within the manuscript.
