# Correction to: *In-vitro* assessment of cutaneous immune responses to *aedes* mosquito salivary gland extract and dengue virus in Cambodian individuals

**DOI:** 10.1093/oxfimm/iqae007

**Published:** 2024-07-26

This is a correction to: David Guerrero, Sokchea Lay, Eakpor Piv, Chansophea Chhin, Sokkeang Leng, Ratana Meng, Kim Eng Mam, Polidy Pean, Amelie Vantaux, Sebastien Boyer, Dorothée Missé, Tineke Cantaert, *In-vitro* assessment of cutaneous immune responses to *aedes* mosquito salivary gland extract and dengue virus in Cambodian individuals, *Oxford Open Immunology*, Volume 5, Issue 1, 2024, iqae003, https://doi.org/10.1093/oxfimm/iqae003

In the originally published version of this manuscript, the reviewer reports were inadvertently omitted from the supplementary material. These have been restored.

