# Supplementary material for: In-vitro assessment of cutaneous immune responses to aedes mosquito salivary gland extract and dengue virus in Cambodian individuals
Source: Oxf Open Immunol. 2024 Apr 1;5(1):iqae003. doi: 10.1093/oxfimm/iqae003 (PMC11035005; doi:10.1093/oxfimm/iqae003)
Supplement: iqae003_Supplementary_Data [file iqae003_supplementary_data.docx]

**Supplementary figures and tables**

| **Innate Panel** | | | |
| --- | --- | --- | --- |
| **Fluorochrome** | **Marker** | **Clone** | **Company** |
| BUV496 | HLA-DR | TU39 | BD biosciences |
| BUV737 | CD16 | 3G8 | BD biosciences |
| BV605 | CD14 | 63D3 | BioLegend |
| BV650 | CD1c | L161 | BioLegend |
| BV711 | CD192 (CCR2) | K036C2 | BioLegend |
| BV785 | CD69 | FN50 | BioLegend |
| AF800 | 4G2 (IC) | 4G2 | In house |
| PE-Texas Red | CD45 | HI30 | BioLegend |
| PE | CD207 | 10E2 | BioLegend |
| PE-Cy7 | CD11b | LM2 | BioLegend |
| APC-R700 | CD15 | HI98 | BioLegend |
| Zombie aqua | Viability |  | BioLegend |

| **Adaptive Panel** | | | |
| --- | --- | --- | --- |
| **Fluorochrome** | **Marker** | **Clone** | **Company** |
| BUV395 | CD3 | SK7 | BD biosciences |
| BUV496 | CD4 | SK3 | BD biosciences |
| APC | HLA-DR | LN3 | BioLegend |
| BV650 | CCR4 | 1G1 | BD biosciences |
| BV711 | CXCR3 | G025H7 | BioLegend |
| BV785 | CD69 | FN50 | BioLegend |
| PE-Texas Red | CD45 | HI30 | BioLegend |
| PE | CD152 (CTLA-4) | HI30 | BioLegend |
| PE-Cy7 | PD-1 | EH12.1 | BD biosciences |
| APC-R700 | CD25 | 2A3 | BD biosciences |
| APC-H7 | CD8 | SK1 | BD biosciences |
| Zombie aqua | Viability |  | BioLegend |

**Supplementary Table 1.** Antibody panels used in the study.


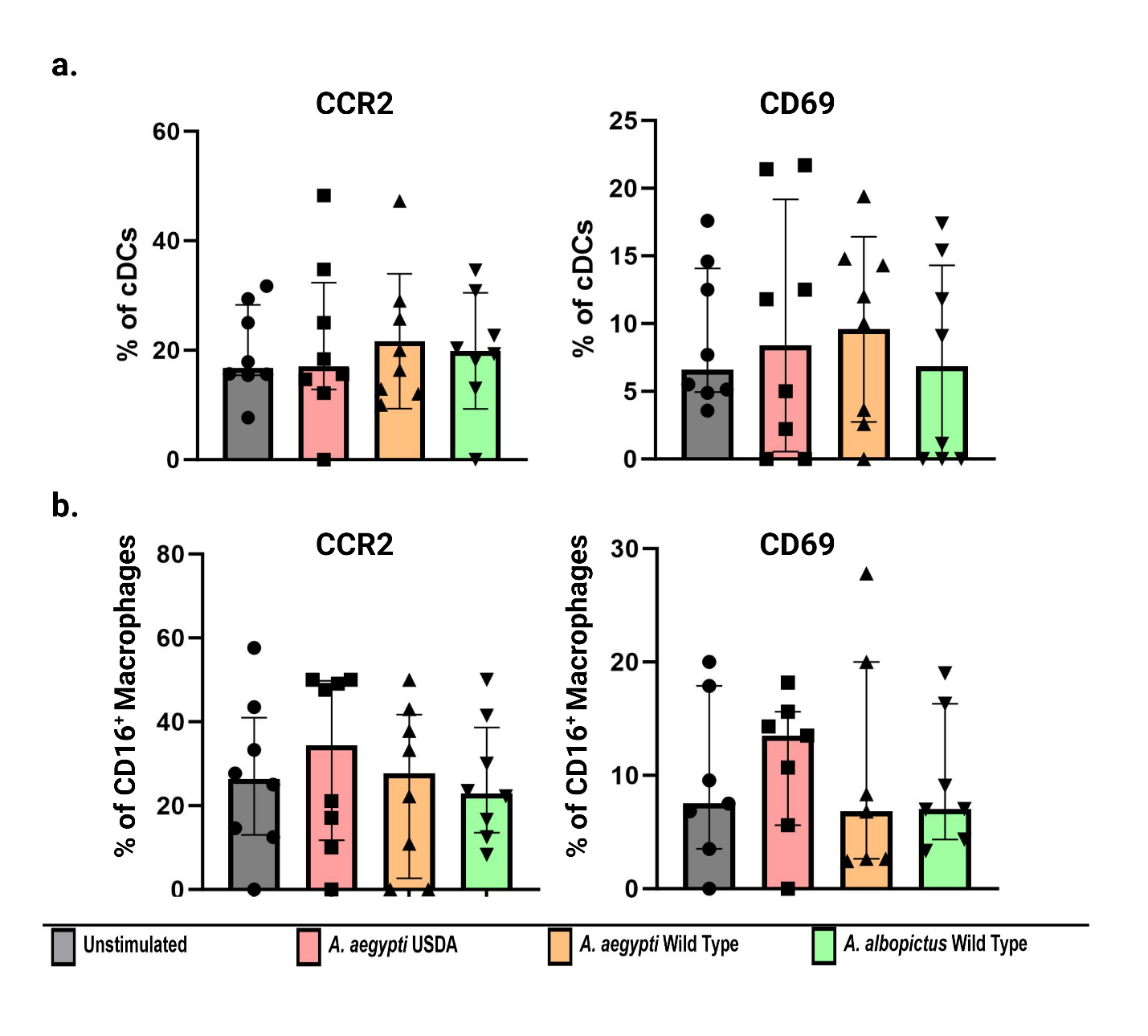


**Supplementary figure 1. Activation marker expression on dendritic cells (CD45+CD1c+) and CD16^+^ macrophages (CD45+CD14+ CD16+) after SGE treatment.** Cells exposed to SGE for 48 hours. No significant changes in the proportion of CD69^+^ nor CCR2^+^ **a.** Dendritic cells (CD45+CD1c+) nor **b.** Macrophages (CD45+CD14+ CD16+). Statistical analysis were performed with Wilcoxon signed-rank test two tailed comparing the unstimulated condition to each of the different stimulated conditions. Bars indicate median and interquartile range. Grey: unstimulated condition, Red: SGE from *A. aegypti* USDA strain, Yellow: SGE from *A. aegypti* WT strain, Green: SGE from *A. albopictus* WT strain. N = 8 individuals. *p<0.05


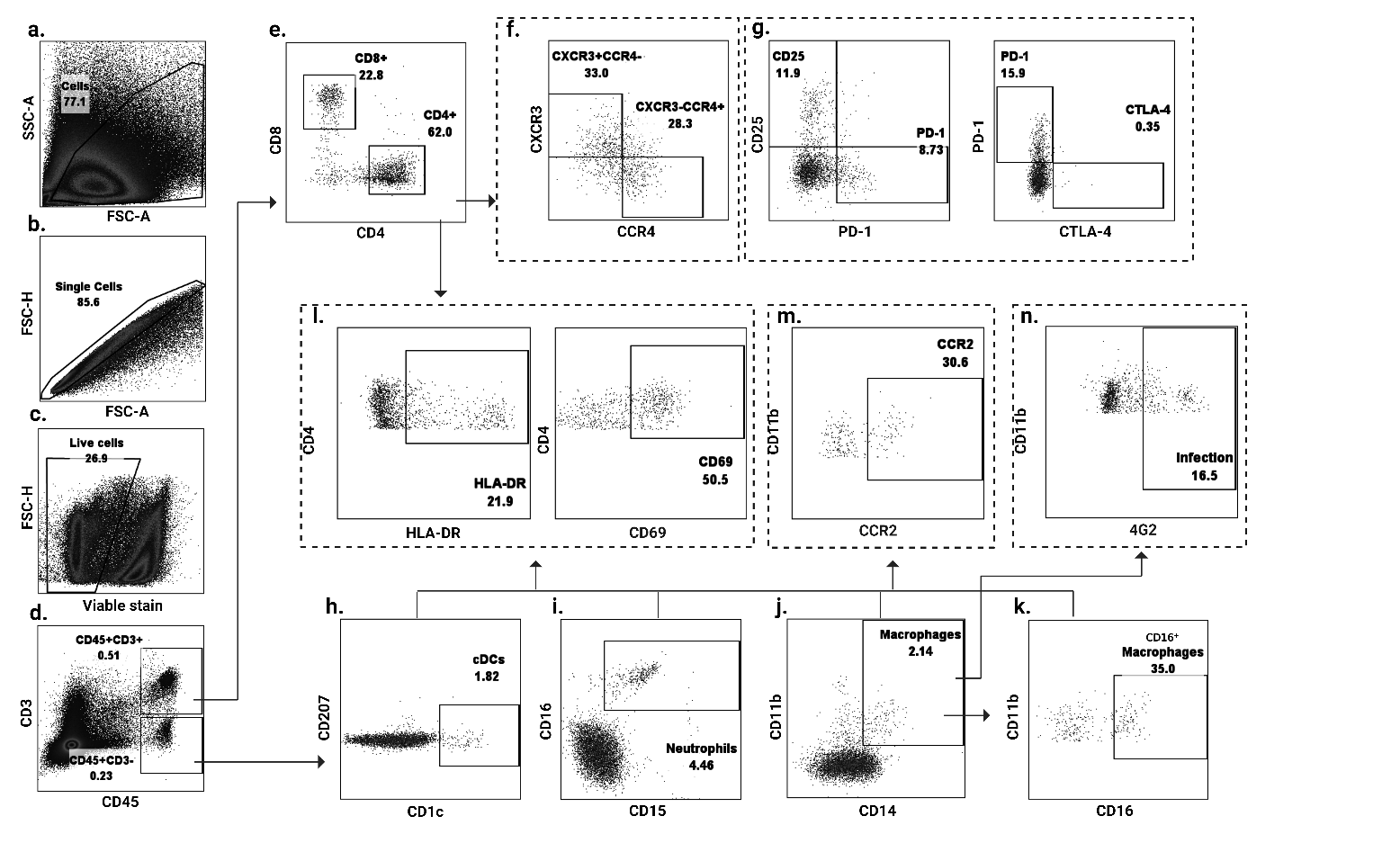


**Supplementary figure 2.** Flow cytometry gating strategy **a**. All cells. **b.** Singlets **c.** Live cells **d.** CD3^+^ and CD45^+^ gates **e.** CD8^+^ and CD4^+^ T cells gates **f.** Th17/Th2 (CD4^+^CCR4^+^CXCR3^-^) and Th17/Th1 (CD4^+^CCR4^-^ CXCR3^+^) compartments **g**. activation markers CD25, PD-1 and CTLA-4 were evaluated both on CD4+ and CD8+ cells **h.** Langerhans cells (CD207^+^) dermal DCs (CD1c^+^) **i.** Neutrophils (CD15^+^CD16^+^) **j.** Macrophages (CD11b^+^CD14^+^) **k.** CD16**^+^** Macrophages (CD11b^+^CD14^+^CD16^+^) **l.** Activation markers HLA-DR and CD69 were evaluated on dermal DCs (CD1c^+^), Neutrophils (CD15^+^CD16^+^), Macrophages (CD11b^+^CD14^+^), CD16^+^ Macrophages (CD11b^+^CD14^+^CD16^+^) and CD8^+^ and CD4^+^ T cells **m.** CCR2 marker evaluated on dermal DCs (CD1c^+^), Neutrophils (CD15^+^CD16^+^), Macrophages (CD11b^+^CD14^+^), CD16^+^ Macrophages (CD11b^+^CD14^+^CD16^+^) **n.** Marker for DENV infection anti-DENV E protein clone 4G2.


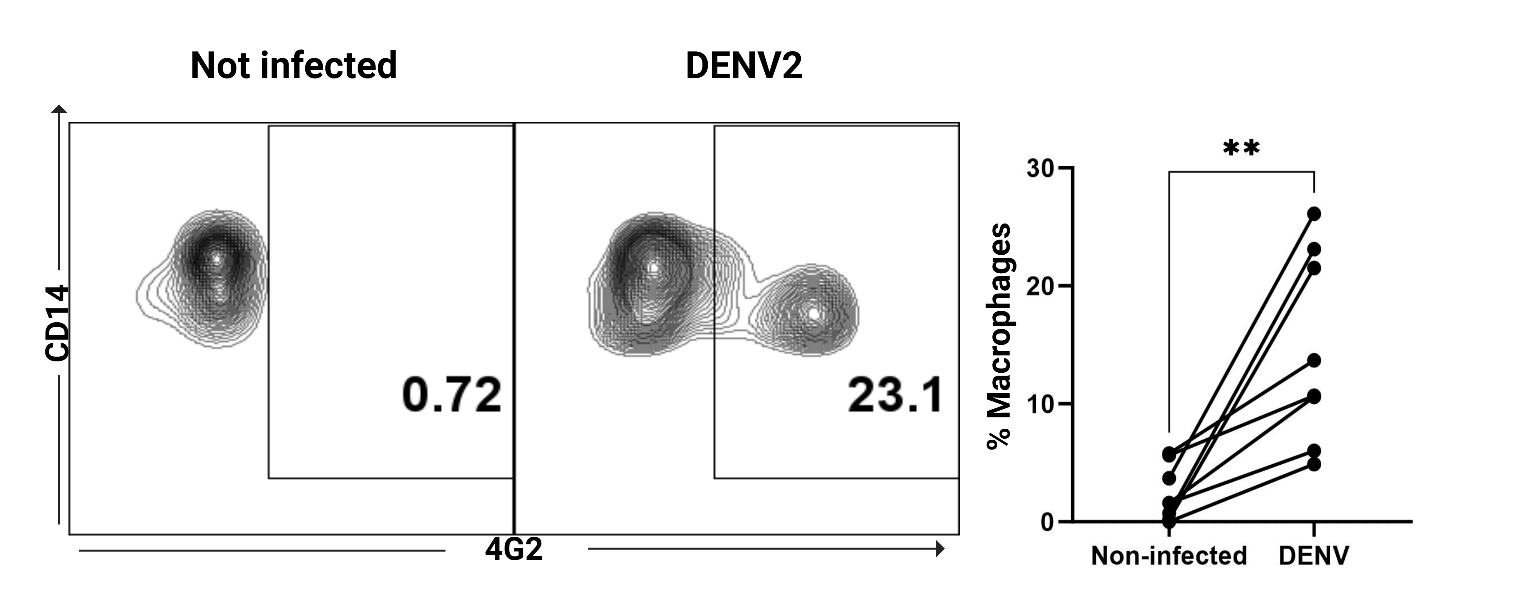


**Supplementary figure 3.** Confirmation of DENV infection on Macrophages. Statistical analyzes were performed with Wilcoxon signed-rank test. N = 8 individuals. *p<0.05
